# Identification of Two Critical Contact Residues in a Pathogenic Epitope from Tetranectin for Monoclonal Antibody Binding and Preparation of Single-Chain Variable Fragments

**DOI:** 10.3390/biom15081100

**Published:** 2025-07-30

**Authors:** Juncheng Wang, Meng Liu, Rukhshan Zahid, Wenjie Zhang, Zecheng Cai, Yan Liang, Die Li, Jiasheng Hao, Yuekang Xu

**Affiliations:** 1 Xinjiang Key Laboratory of Biological Resources and Genetic Engineering, College of Life Science and Technology, Xinjiang University, Urumqi 830017, China; 2131010072@ahnu.edu.cn; 2 Anhui Provincial Key Laboratory for Conservation and Exploitation of Biological Resources, College of Life Sciences, Anhui Normal University, Wuhu 241000, China; 2121011631@ahnu.edu.cn (M.L.); rukhshanzahid@ahnu.edu.cn (R.Z.); zhangwenjie@yjsyy.com (W.Z.); 2221011740@ahnu.edu.cn (Z.C.); 2321011720@ahnu.edu.cn (Y.L.); 2321021782@ahnu.edu.cn (D.L.); jiashenghao123@ahnu.edu.cn (J.H.); 3 School of Basic Medicine, Nanchang Medical College, Nanchang 330052, China; 4 The First Affiliated Hospital of Wannan Medical College, Wuhu 241001, China

**Keywords:** sepsis, tetranectin, P5-5, epitope, scFv

## Abstract

Sepsis is a fetal disease that requires a clear diagnostic biomarker for timely antibiotic treatment. Recent research has identified a pyroptosis-inducing epitope known as P5-5 in tetranectin (TN), a plasma protein produced by monocytes. Previously, we produced a 12F1 monoclonal antibody against the P5-5 and discovered that it could not only diagnose the presence but also monitor the progress of sepsis in the clinic. In the current study, we further investigated the structure site of the P5-5 and the recognition mechanism between the 12F1 mAb and the P5-5 epitope. To this end, 10 amino acids (NDALYEYLRQ) in the P5-5 were individually mutated to alanine, and their binding to the mAb was tested to confirm the most significant antigenic recognition sites. In the meanwhile, the spatial conformation of 12F1 mAb variable regions was modeled, and the molecular recognition mechanisms in detail of the mAb to the P5-5 epitope were further studied by molecular docking. Following epitope prediction and experimental verification, we demonstrated that the motif “DALYEYL” in the epitope sequence position 2−8 of TN-P5-5 is the major binding region for mAb recognition, in which two residues (4L and 8L) were essential for the interaction between the P5-5 epitope and the 12F1 mAb. Therefore, our study greatly narrowed down the previously reported motif from ten to seven amino acids and identified two Leu as critical contact residues. Finally, a single-chain variable fragment (scFv) from the 12F1 hybridoma was constructed, and it was confirmed that the identified motif and residues are prerequisites for the strong binding between P5-5 and 12F1. Altogether, the data of the present work could serve as a theoretic guide for the clinical design of biosynthetic drugs by artificial intelligence to treat sepsis.

## 1. Introduction

Sepsis, a life-threatening organ dysfunction caused by a dysregulated host response to infection, represents a major worldwide health burden with an estimated annual incidence of 48.9 million cases and 11 million fatalities [1,2,3,4,5]. As pathogens are the predominant cause of sepsis, prompt and accurate diagnosis is crucial due to the rapid disease progression and quick elapse of a critical “golden window” for effective intervention [6]. However, current diagnostic and prognostic approaches face significant limitations. While microbial culture remains the gold standard for sepsis diagnosis, its clinical utility is hampered by a prolonged turnaround time of 2–3 days, delaying timely therapeutic decisions [7]. Rapid alternatives such as C-reactive protein (CRP), procalcitonin (PCT), and interleukin-6 (IL-6) testing provide faster results but suffer from insufficient specificity, often leading to unnecessary broad-spectrum antibiotic use [8,9]. This contributes to antimicrobial resistance (AMR), increased treatment costs, and potential adverse patient outcomes [1].

Furthermore, sepsis prognosis currently relies on clinical scoring systems like the Sequential Organ Failure Assessment (SOFA) and Quick SOFA (qSOFA), which, despite being widely adopted, exhibit notable complexity and misdiagnosis rates of up to 30% [1,10]. The empirical determination of antibiotic therapy duration in sepsis management further exacerbates the risk of antibiotic overuse and AMR. Given these challenges, there is an urgent need for more reliable biomarkers and rapid diagnostic methods to improve sepsis detection, prognostic accuracy, and antimicrobial stewardship, ultimately enhancing patient outcomes in critical care settings.

Recent research revealed that certain plasma and tissue proteins, notably tetranectin (TN), are found in the plasma of patients with sepsis or septic shock [11,12]. TN was discovered in 1986 as an oligomeric fibrinogen-binding protein mostly expressed in the lungs. Further analysis of patients and healthy controls showed that plasma TN levels were reduced by 60–70% in patients with sepsis or septic shock. Consistent with these clinical findings, there was a corresponding reduction in circulating TN levels in experimental sepsis, with a reduction of more than 70% by 24 h after the onset of disease, which is the time point at which some septic animals begin to die. Mechanistically, TN was involved in the pathogen-induced pyroptosis of macrophages for the development of sepsis [13]. Importantly, a crucial epitope in the structure of TN proteins is P5-5, which mediates the interaction of TN with high mobility group box 1 (HMGB1), a protein released by activated leukocytes. Phagocytosis of the TN/HMGB1 complex by macrophages promoted their pyroptosis. leading to significant reduction in macrophage number and depletion of plasma TN levels in septic patients [11]. Therefore, if TN can be utilized as an immunological biomarker for sepsis, the P5-5 epitope in the TN holds promise for quicker clinical diagnosis and symptom assessment of the disease [11,12].

In our previous study, we generated a hybridoma cell line targeting P5-5 and successfully purified the monoclonal antibody 12F1 [14]. Functional analyses revealed that this antibody not only facilitates the assessment of disease severity in patients but also exhibits therapeutic potential by attenuating sepsis progression in murine models [14]. Until now, we only know that the pathogenic epitope P5-5 is composed of 10 amino acids (NDALYEYLRQ) [11]. However, which of the 10 amino acids are the critical residues to mediate the interaction with the therapeutic antibodies is far from clear. This study aims to identify the key contact residues in the pathogenic P5-5 epitope through antigen design and molecular docking. Furthermore, site-directed mutagenesis was employed to elucidate the detailed interactions between P5-5 and the mAb. The effect of the antigen linear epitope on its interaction with the antibody, as well as the mAb’s binding mechanism with the pathogenic epitope, was explored and characterized based on the epitope information. Finally, the amplified VH and VL genes, which encode antibodies against P5-5, were employed to create an scFv, and the recombinant antibody was generated through prokaryotic expression to validate our results.

## 2. Materials and Methods

### 2.1. Materials

Regal Biology Technology (Shanghai, China) provided the bovine serum albumin (CAS: 9048-46-8) for this study. Sigma Aldrich (St. Lois, MO, USA) supplied ovalbumin (OVA, CAS: A5378), an albumin derived from chicken egg white. The P5-5 epitope (Homo tetranectin peptide NDALYEYLRQ) and mutants (ADALYEYLRQ, NAALYEYLRQ, NDAAYEYLRQ, NDALAEYLRQ, NDALYAYLRQ, NDALYEALRQ, NDALYEYARQ, NDALYEYLAQ, NDALYEYLRA) were synthesized and bought from Sangon (Shanghai, China). HPLC validated the purity of 10 peptides at ≥95.5%. Furthermore, 6 × His, mouse anti-His-Tag Monoclonal antibody (CAS: No. 66005-1-Ig), and rabbit anti-MBP (Maltose binding protein)-Tag Polyclonal antibody (CAS: No. 15089-1-AP) were sourced from Proteintech (Wuhan, China). HRP conjugate and goat anti-mouse IgG (H + L) (CAS: BF03001) and Goat anti-rabbit IgG conjugated with HRP (CAS: BF03008) were acquired from Biodragon (Suzhou, China). Protein G agarose beads for affinity chromatography (CAS: L00209) were obtained from GenScript (Nanjing, China). All other reagents and substances were of analytical grade. They were obtained from regular sources.

### 2.2. TN-P5-5 Complete Antigen Preparation

The P5-5 and mutants were conjugated to BSA by the EDC conjugating method. Firstly, the P5-5 and other 9 mutant peptide solutions (4 mg/mL in 0.1 M MES buffer, pH 4.7) were separately mixed with BSA solution (10 mg/mL in 0.1 M MES buffer, pH 4.7). Then, the reaction mixtures were all supplemented with 100 μL of EDC solution (10 mg/mL in deionized water) and incubated at 25 °C for 2 h to facilitate conjugation. The haptens were coupled to BSA at a molar ratio of 10:1 [14]. After a two-hour reaction, the resulting mixtures were subjected to dialysis against 0.01 M phosphate-buffered saline (PBS, pH 7.4) for 48 h. The final products were then stored at −20 °C for the following applications. The P5-5 and mutant conjugates were examined using native gel electrophoresis, and the concentration of the complete antigen was determined using the BCA protein assay kit.

### 2.3. Epitope Prediction of P5-5

The putative epitope location of the P5-5 peptide was further described by B-cell linear epitope prediction analysis using the Immune Epitope Database Analysis Resource service (http://tools.immuneepitope.org/bcell/, accessed on 25 April 2025) [15,16]. Concurrently, the three-dimensional structure of P5-5 was predicted via homology modeling employing the SWISS-MODEL workspace (https://swissmodel.expasy.org/, accessed on 25 April 2025), a web-based framework for modeling protein structural homology [17].

### 2.4. Sequence Analysis of 12F1 Monoclonal Antibody Variable Regions

Total RNA from mAb 12F1 hybridoma cells was extracted with 1 mL TRIzol Reagent and reverse-transcribed to cDNA using the cDNA Synthesis Kit. Azenta used murine IgG-specific primers and NGS sequencing to acquire the mAb 12F1 genes from the heavy and light chain variable regions (VH and VL, respectively). Antibody sequences were analyzed using the NCBI Ig BLAST (v1.17.0) software to identify the most closely matched germline V-D-J gene sequences within the CDR1, CDR2, and CDR3 regions aligned from the IMGT (International Immunogenetics Information System) database (IMGT, https://www.imgt.org/, accessed on 25 April 2025) [18].

### 2.5. 12F1 Monoclonal Antibody Homology Model and Docking with P5-5 Epitope

The three-dimensional structure model of scFv based on monoclonal antibody 12F1 was constructed using the SWISS-MODEL homology modeling method. Among the developed models, the one with the highest rank and quality, as measured by the lowest probability density function energy and the highest discrete optimized protein energy, was chosen for the antibody conformation. The validity of the resulting homology model was assessed through Profile-3D and Ramachandran plot analyses. To optimize the complementarity-determining regions (CDRs), the published antibody crystal structures were compared via the IMGT/V-QUEST database (http://www.imgt.org) [19]. To determine the contact forces and critical amino acid positions, the anticipated P5-5 epitope molecules were docked into the cavity produced by the CDR regions in the active conformation utilizing the ZDock device (ZDOCK server, University of Massachusetts Medical School, Boston, MA, USA) [16,20]. The docking outcomes were visualized using the PyMOL program (version 2.2) [21].

### 2.6. Purification of 12F1 mAb

We previously generated the hybridoma cell line 12F1 in our lab. This cell line stably secreted anti-TN-P5-5 mAb [14]. Antibodies were produced in bulk via in vivo induction in mice to generate ascites. Female BALB/c mice aged 10 weeks bought from Nanjing Junke Bioengineering Ltd. (Nanjing, China) were sensitized by injecting ascites adjuvant into their abdominal cavity. To induce ascites formation, roughly 10^6^ positive hybridoma cells were injected into the abdominal cavities of the mice [22,23,24,25,26,27,28]. All animal procedures were approved by the Animal Ethics Committee of Anhui Normal University (AHNU-ET2022015), and the 3R principles (Replacement, Reduction, Refinement) were followed. The antibodies were then concentrated and purified from the ascitic fluid using Protein G Resin [16]. Afterwards, we used a BCA protein assay kit to measure the amount of purified antibodies and iELISA to assess the titer [22].

### 2.7. TN-P5-5 Mutants Design and Synthesis

Based on the research findings regarding antibody binding to specific epitopes, the key residues involved in the interaction between mAb-P5-5 and its epitope were further identified. A total of 10 amino acids within the TN-P5-5 pathogenic epitope were individually mutated to alanine, and these residues were considered critical for the specific antigen–antibody interaction. By sequentially mutating the residues at positions 1, 2, 4, 5, 6, 7, 8, 9, and 10 to alanine, the corresponding mutated protein sequences—ADALYEYLRQ, NAALYEYLRQ, NDAAYEYLRQ, NDALAEYLRQ, NDALYAYLRQ, NDALYEALRQ, NDALYEYARQ, NDALYEYLAQ, and NDALYEYLRA—were successfully generated.

### 2.8. iELISA and icELISA to Identify the Specific Sites Between 12F1 mAb and TN-P5-5 Interaction

The molecular recognition mechanism of monoclonal antibody 12F1 for P5-5 was determined via two types of ELISA assays [15,16].

Indirect ELISA (iELISA): To identify the key binding epitopes of the prepared mAb to the P5-5 epitope, P5-5-BSA, and P5-5 mutant-BSA, the antigens were coated onto 96-well plates at a concentration of 10 µg/mL [15]. Subsequently, purified 12F1 mAb diluted at 1:2000 in blocking buffer was added to the wells after blocking and washing. Following another washing step, goat anti-mouse IgG-HRP at a 1:5000 dilution in blocking buffer was added and incubated at 37 °C for 1 h. To commence color development, 100 μL of 3,3,5,5-Tetramethylbenzidine (TMB) solution was added to each well, followed by 100 μL of 2 M H_2_SO_4_. The absorbance at 450 nm was measured using a microplate reader.

Indirect competitive ELISA (ic-ELISA): the inhibition ability of 12F1 mAb against P5-5 and its mutants was assessed through icELISA [16]. A coating antigen P5-5-OVA solution at a concentration of 5 μg/mL was prepared and dispensed (100 μL) into each well of a 96-well ELISA plate. The plates were then left to incubate at 4 °C overnight. Following washing, 5% (*w*/*v*) PBSM was added to each well and incubated at 37 °C for 2 h. The synthesized peptides of P5-5 and its variants were prepared as 1 mg/mL stock solution (dissolved in 0.01M PBS, pH 7.4). These peptides were then serially diluted in PBSM to concentrations of 20 μg/mL, 10 μg/mL, 5 μg/mL, 2.5 μg/mL, 1.25 μg/mL, 625 ng/mL, 312.5 ng/mL, 156.25 ng/mL, 78.13 ng/mL, 39.06 ng/mL, 19.53 ng/mL, 9.77 ng/mL, 4.88 ng/mL, 2.44 ng/mL, 1.22 ng/mL, and 0.61 ng/mL for the competitor in the ic-ELISA assay [29]. Subsequently, 12F1 mAb was mixed thoroughly with the diluted solutions of P5-5 and its mutants at equal volume at 37 °C for 30 min incubation and reaction. Following this, each well was impressed by 100 μL of the mixture, which was then incubated at 37 °C for one hour. The succeeding stages, which included the addition of HRP-goat anti-mouse serum, TMB substrate solution, and stop solution, were carried out as described earlier. The measurement of the OD value was then taken under 450 nm. The IC_50_ values of the 12F1 monoclonal antibody against synthesized P5-5 and nine other mutant peptides were determined using the four-parameter logistic equations in Prism 9.0 GraphPad software (version 9.0 GraphPad Software), following the established methodology.

### 2.9. Construction of the MBP Fusion scFv Antibody, Soluble Expression, and Purification

Specific primers were designed to amplify the target scFv gene after sequencing the 12F1 hybridoma clone. The pMAL-c2x vector was utilized to create expression vectors containing the scFv protein fused with an MBP tag. The recombinant pMAL-c2X-scFv plasmid was transformed into *E. coli* TB1 through electroporation for scFv expression. To induce the fused scFv expression, 1 mM of isopropyl β-D-thiogalactopyranoside (IPTG) was added to the LB culture overnight at 16 °C until the OD600 reached 0.8 [30]. Subsequently, cell pellets were collected by centrifugation at 4 °C and subjected to ultrasonic fragmentation. The purification of the expressed anti-P5-5 scFv was performed through MBP affinity chromatography, and the resulting proteins were evaluated using SDS-PAGE [31].

### 2.10. Binding Activity Determination of Anti-P5-5 scFv by iELISA

The evaluation of the binding activity of purified scFv was conducted using the indirect ELISA protocol as described in 2.8. In brief, ELISA plates were coated overnight at 4 °C with serially diluted OVA-P5-5 conjugate antigen. Plates were washed three times with PBST (0.05% Tween-20 in PBS), then blocked with 5% (*w*/*v*) skimmed milk in PBS for two hours at 37 °C. Subsequently, purified scFv fusion proteins at varying concentrations were added to triplicate wells and incubated at 37 °C for two hours. Following subsequent washing processes, a mouse anti-MBP tag primary antibody (1:1000 dilution) was used to identify specific binding, which was then incubated for 1 h at 37 °C with an HRP-conjugated goat anti-mouse IgG secondary antibody (1:5000 dilution). The enzymatic reaction started with a tetramethylbenzidine (TMB) substrate solution and ended after 15 min with a 2 M H_2_SO_4_ stop solution. Optical density measurements were obtained under 450 nm using a microplate reader. The affinity constant Kaff was calculated based on ELISA absorbance. This procedure involved equal dilutions of the coated antigen and the antibody. Kaff was then determined using the equation below. The ratio of [Ag] to [Agt] was denoted as n, where n = [Ag]/[Agt] and [Ag] and [Ag]t denote the concentrations of the coated antigen. The K_aff_ formula calculated was K_aff_ = (n − 1)/{2(n[Abt] − [Ab])} [32,33]. [Ab]t and [Ab] denote the concentration of the scFv at 50% of its OD max [33,34,35].

### 2.11. The Sensitivity Determination of Anti-P5-5 scFv

To evaluate the antibody sensitivity to the P5-5 epitope, we conducted ic-ELISA. The competitive antigen (standard P5-5 epitope) was incubated with purified MBP-linker-scFv fusion protein at 37 °C for 2 h [16]. Subsequent steps followed the standard ELISA protocol outlined in Section 2.7. Data analysis was performed using Origin 8.0 software to generate a four-parameter logistic curve. The binding ratio (B/B0) was calculated by analyzing the ratio of antibody binding in the presence versus absence of competing antigen across a concentration gradient of P5-5 [15].

### 2.12. Statistical Analysis

Experimental datasets were subjected to comprehensive processing and statistical analysis utilizing Origin 8.0 and GraphPad Prism 9.0. The analytical workflow incorporated systematic curve-fitting procedures complemented by analysis of variance (ANOVA) to characterize variable relationships. All experimental determinations were conducted with rigorous replication, maintaining a minimum of three independent experimental replicates for each dataset [36,37].

## 3. Results

### 3.1. Epitope Binding Pattern Prediction

**Figure 1:**
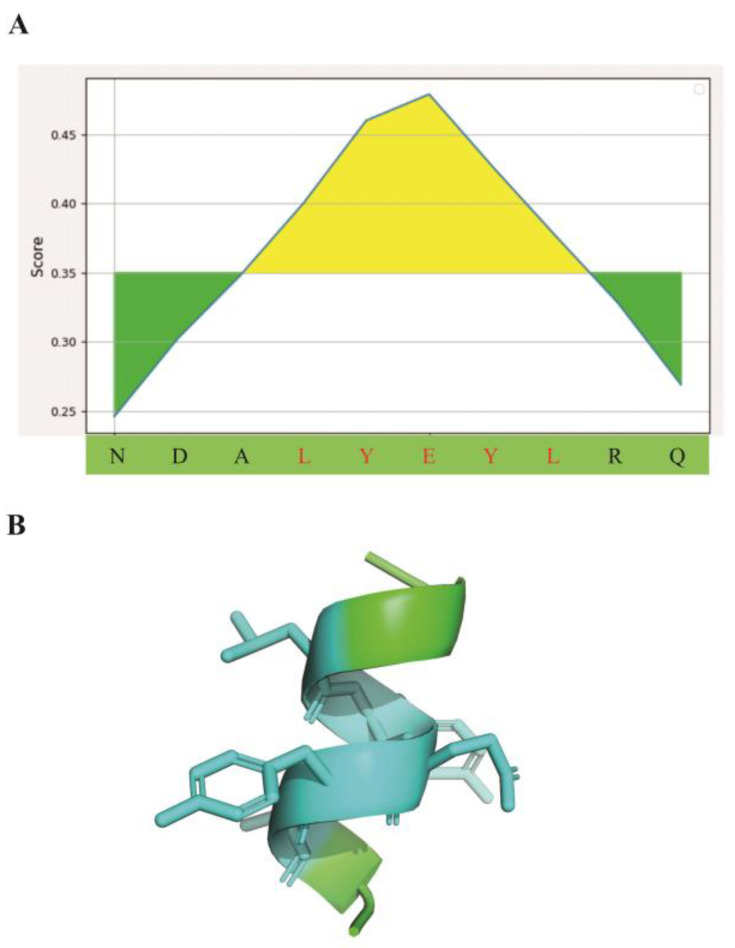
TN-P5-5 epitope prediction and three-dimensional structural analysis. (**A**) The output graph of presented linear B-cell epitopes displays amino acid positions (X-axis) and Bepipred scores (Y-axis). Residues with scores above the default threshold of 0.35 (shown in yellow) are more likely to be part of the epitope. Green implies a decreased likelihood of belonging to the epitope. (**B**) The 3D structure of TN-P5-5 with the predicted epitope from SWISS-MODEL homology modeling. Amino acid residues 4–8 motif (Leu-Tyr-Glu-Tyr-Leu) are shown in light blue.

To analyze the critical contact residues in the pathogenic P5-5 epitope, a 10-mer peptide with the sequence of NDALYEYLRQ in TN protein, we first utilized the Bepipred-2.0 algorithm for B-cell linear epitope prediction and identified prominent linear epitopes spanning residues 4–8 (motif Leu-Tyr-Glu-Tyr-Leu) within the peptide sequence (Figure 1A). The epitope structure of P5-5 is predicted to consist of an α helix and a binding site covering linear amino acid sequences Leu4 to Leu8 for antibody recognition in three-dimensional conformation (Figure 1B). Collectively, the small P5-5 sequence demonstrated distinct immunogenic properties in comprehensive epitope mapping.

To be specific, Figure 1B illustrates the three-dimensional structure of P5-5 within the native TN protein, aiming to depict the spatial arrangement of the linear epitope sequence in its original context. The TN-P5-5 amino acid sequence consists of 10 residues, and its corresponding structural conformation is characterized as an α-helix. The antigenic epitope of P5-5 was predicted using the BepiPred B-cell linear epitope prediction tool (http://tools.immuneepitope.org/bcell/, accessed on 25 April 2025). The core amino acid sequence (positions 4–8) of this linear epitope was identified as “LYEYL” (Figure 1A), and its spatial location corresponds to the α-helix region (Figure 1B). In the subsequent experimental phase of this study, the epitope-specific monoclonal antibody was validated through chemical synthesis of the corresponding peptide epitope [15]. The entire 10-amino acid sequence of P5-5, including the LYEYL motif, was synthesized as a linear peptide, which serves as a linear epitope.

### 3.2. Antibody Variable Region Structure Prediction of 12F1 and Molecular Docking with P5-5 Epitope

**Figure 2:**
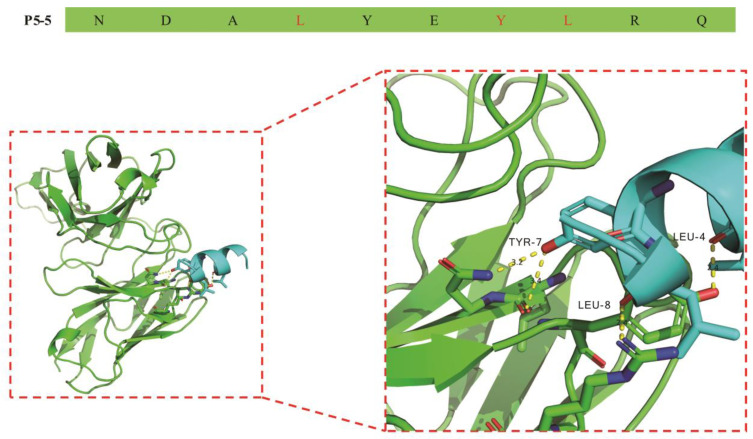
12F1 mAb homology model and molecular dock. The scFv binding location and interactions between the antibody paratope and the P5-5 epitope were revealed following docking. The conformation prediction of scFv residues (green) to the TN-P5-5 epitope (blue) was depicted as a stick model and labeled. Among them, residues of P5-5 involved in the binding pockets are Leu4, Tyr7, and Leu8.

With the antigenic information of the targeted peptide in hand, we next turned to its interacting antibody. We began by generating the antibody’s cDNA chain via RT-PCR employing total RNA obtained from 12F1 hybridoma cells as the template. Subsequently, the cDNA template was utilized to amplify the antibody gene. The 3D conformation of mAb 12F1 was then precisely predicted by matching its amino acid sequence to reported protein structures in the PDB database. The antibody variable region was subsequently docked with the P5-5 epitope using the ZDOCK online server. As depicted in Figure 2, it was observed that the P5-5 epitope conformation resided within the binding cavity of the murine 12F1 mAb. Moreover, examination of the antigen–antibody interaction complex revealed formation of hydrogen bonds between the residues of the murine antibody and the P5-5 epitope. Specifically, a Leu4 residue in P5-5 and a Tyr184 residue in the mAb were found to establish a hydrogen bond with a bond length of 2.4 Å. Additionally, the Tyr7 residue in P5-5 interacted with two residues (Leu167 and Asn168) in the antibody through hydrogen bonds at distances of 3.40 Å and 3.20 Å, respectively, which are important in the antibody–antigen interaction. Furthermore, a hydrogen bond (2.4 Å) was formed between Leu8 in P5-5 and Arg187 of the antibody. Overall, these four hydrogen bonds were identified as the primarily sensitive and specific binding forces for antibody recognition, thereby enhancing the stability and specificity of the complex.

### 3.3. Molecular Recognition Mechanism of mAb 12F1 to P5-5

**Figure 3:**
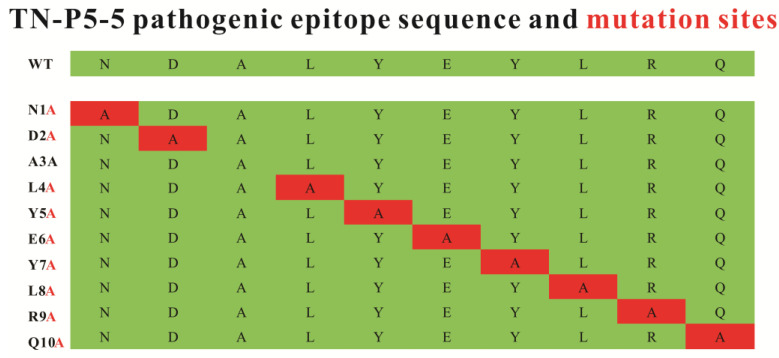
TN-P5-5 pathogenic epitope sequence and mutagenesis map of the P5-5 epitope through alanine (red) scan. To find out the interaction of the 12F1 mAb and different mutants of P5-5 recognition, standard peptides of ADALYEYLRQ, NDALYEYLRQ, NAALYEYLRQ, NDAAYEYLRQ, NDALAEYLRQ, NDALYAYLRQ, NDALYEALRQ, NDALYEYARQ, NDALYEYLAQ, and NDALYEYLRA were chemically synthesized.

Following theoretic prediction based on the amino acid sequence and their conformational structure, site-directed mutagenesis was performed to determine the critical contact residues in the P5-5 epitope and their mechanism of interaction with the 12F1 mAb. Since alanine is a type of hydrophobic amino acid lacking side groups and has a relatively simple structure, alanine scanning constitutes an extremely effective approach for investigating the key amino acids within the interaction region of antibody and antigen [16,17]. By mutating through alanine scanning and comparing the mutated P5-5 epitope with its reactive mAb in binding affinity, the determination of the amino acid residues and their position to interact with its antibody becomes feasible. Figure 3 depicts the mutant designs, with “A” indicating the locations that were mutated to alanine to scan the critical amino acids involved for binding activity.

**Figure 4:**
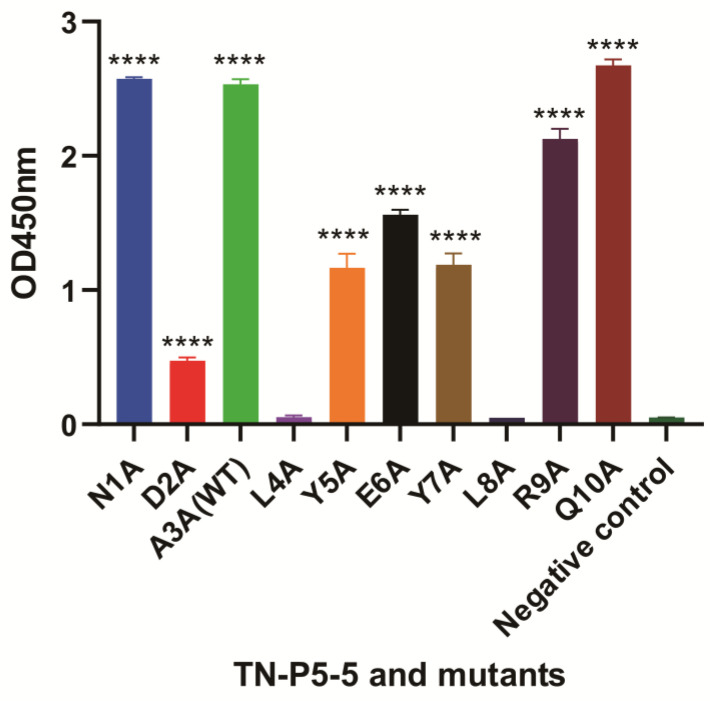
Epitope identification based on P5-5 (WT) and mutants. The indirect ELISA titer for various site mutant peptides. Error bars represent the standard deviation (SD) of six repeated experiments. Data are presented as mean ± SD. **** *p* < 0.0001.

**Figure 5:**
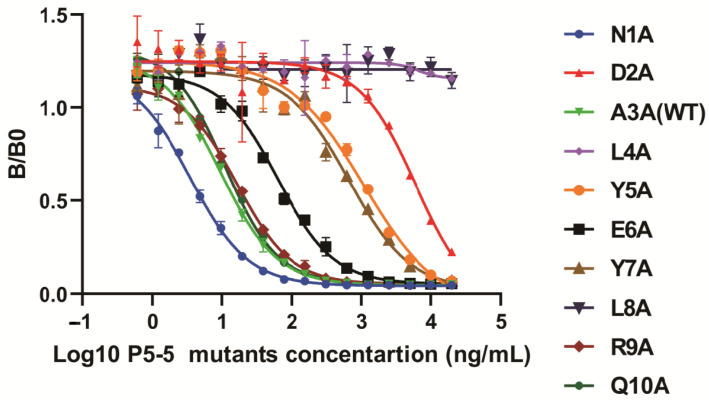
Molecular recognition mechanism of 12F1 mAb towards TN-P5-5. Competitive reactions of P5-5 and mutagenesis. The concentrations of P5-5 (WT) and mutagenesis were diluted to 20 μg/mL, 10 μg/mL, 5 μg/mL, 2.5 μg/mL, 1.25 μg/mL, 625 ng/mL, 312.5 ng/mL, 156.25 ng/mL, 78.13 ng/mL, 39.06 ng/mL, 19.53 ng/mL, 9.77 ng/mL, 4.88 ng/mL, 2.44 ng/mL, 1.22 ng/mL, and 0.61 ng/mL with blocking solution as competition antigen to react with 12F1 mAb, respectively.

Ascites were obtained from BALB/c mice following the injection of cultured hybridoma 12F1 cells. The anti-TN-P5-5 mAb 12F1 (IgG1) was then extracted from the ascites using affinity chromatography and a protein G resin. SDS-PAGE analysis of purified mAb 12F1 (Figure S1) revealed two different protein bands at 50 kDa and 25 kDa, which correspond to the heavy and light chains. This result demonstrates the effective purification of mAb 12F1. To find out the key regions responsible for 12F1 mAb-TN-P5-5 interactions, nine amino acids at the pathogenic epitope of P5-5, with the exception of A3, were mutated to alanine using site-directed mutagenesis. The detecting antigen P5-5-BSA and other mutants-BSA were coated, and the indirect ELISA results in Figure 4 revealed that the binding activities of 12F1 mAb against two mutated amino acids (L4A and L8A) were all significantly reduced to levels similar to the negative control (carrier protein BSA). Other mutants (DA2, Y5A, E6A, and Y7A) also exhibited weak binding signals to 12F1 mAb recognition compared with the WT P5-5 (Figure 4). Meanwhile, ic-ELISA using P5-5 peptides with various alanine substitutions and the 12F1 monoclonal antibody, respectively, was also performed to evaluate antibody inhibitory capacity against the antigens. The results in Figure 5 were consistent with those obtained from indirect ELISA. Following mutation, L4A and L8A entirely lost their ability to bind antibodies. The antigen inhibitory capabilities of the mutated peptides DA2, Y5A, E6A, and Y7A were significantly reduced compared to the WT P5-5, resulting in partial loss of their antibody-binding affinity (Figure 5). Overall, the results showed that the amino acids L4 and L8 in the pathogenic epitope were the most crucial residues for the interaction of 12F1 mAb and P5-5.

### 3.4. Bioinformatic Analysis of the Anti-P5-5 scFv

Then, we went on to make recombinant anti-P5-5 scFv from the hybridoma firstly to confirm the identified motif and critical contact residues in P5-5 and then preserve the specificity of the valuable theranostic antibody, as hybridoma cell lines may cease antibody production after prolonged cultivation. To achieve this, the VH, VL, and linker genes were assembled into the entire scFv gene via an SOE-PCR (gene splicing by overlap extension PCR) reaction, yielding a 723 bp completed scFv fragment. Figure S2 depicts the scFv’s coding sequence, which contains 241 amino acids and includes a (Gly4Ser)3 linker between VH and VL. The three complementarity-determining regions (CDRs) of VH and VL have been determined based on Kabat’s approach [38], as illustrated in Figure S2. Meanwhile, Figure S3A displayed the scFv IMGT “collier de perle” graphical two-dimensional (2-D) representations. Sequence alignment of various mouse germline genes using multiple bioinformatics resources (IMGT databases) confirmed that the VH and VL sequences of the scFv correspond to the variable regions of mouse antibody genes, as illustrated in Figure S3B.

### 3.5. Expression, Purification, and Identification of Anti-P5-5 scFv

**Figure 6:**
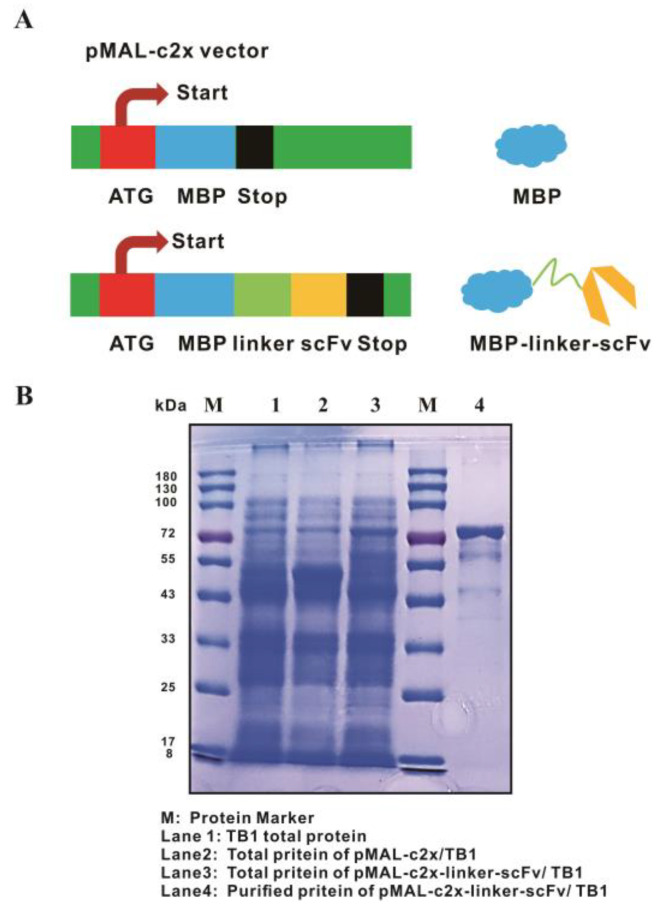
Construction and production of scFv fusion proteins that target TN-P5-5. (**A**) The diagram illustrates the construction of MBP tag and MBP-fused scFv formats against the P5-5 epitope in this study (MBP, MBP-linker-scFv). ATG, start codon; MBP, maltose binding protein; Linker, (G_4_S)_3_ linker chain; scFv, single-chain variable fragment; Stop, stop codon. (**B**) The production and purification of fused scFv proteins were analyzed based on SDS-PAGE. The molecular weight of the expressed MBP protein was approximately 50 kDa, while the presence of distinct protein bands at 70 kDa confirms the successful purification of the scFv following *E. coli* lysis. M: Protein marker; Lane 1: control *E. coli* TB1 proteins; Lane 2: MBP tag protein (empty vector pMAL-c2x in *E. coli* TB1); Lane 3: Expressed total proteins of MBP-linker-scFv; Lane 4: Purified scFv protein.

Following the successful construction of a fusion expression vector, pMAL-c2x-linker-scFv (sequenced and aligned as Figure S4), with the pMAL-c2x vector serving as a control depicted in Figure 6A, we then introduced it into *E. coli* TB1 to express the scFv. Figure 6B illustrated the expression of the scFv fusion proteins in *E. coli* TB1 cells, their purification using MBP affinity resin, and confirmation through SDS-PAGE analysis. The molecular weight of the produced MBP protein was around 50 kDa, while that of the MBP-linker-scFv fusion proteins increased to around 70 kDa [30,39]. To maintain the structural and functional integrity of the fusion proteins, the recombinant scFvs were purified from the induced bacteria using affinity chromatography under gentle conditions, resulting in protein purity exceeding 90% (Figure 6B).

Furthermore, an indirect ELISA was performed to demonstrate the scFv’s affinity and inhibition against the P5-5 antigen. As depicted in Figure S4, the affinity constant of the scFv was determined to be 1.19 × 10^7^ L/mol (Figure S5), indicating a high-affinity antibody (typically defined as an affinity falling within the range of 10^7^ to 10^12^ L/mol). A standard curve for the indirect competitive ELISA (ic-ELISA) targeting the P5-5 epitope was established by utilizing varying concentrations of the P5-5 standard peptide as a competing antigen to interact with the purified MBP-linker-scFv fusion proteins (Figure S6). The relationship between the concentrations of P5-5 and the inhibition value was analyzed using Origin 8 software. The half-maximal inhibitory concentration (IC_50_) of P5-5 binding to the scFv was calculated to be 359.76 ng/mL (Figure S6A). Moreover, our existing ic-ELISA methods may be used to analyze P5-5 epitope samples in PBS, with a target detection range of 78.125 ng/mL to 1.25 μg/mL (Figure S6B), and the limit of detection (LOD) was 62.86 ng/mL. Collectively, these findings suggest that the expressed and purified MBP-Linker-scFvs may recognize the same motif and exhibit functionalities akin to those of their parental 12F1 monoclonal antibody.

## 4. Discussion

Studies have demonstrated that C-reactive protein, procalcitonin, tumor necrosis factor α, interleukin-6, and neutrophil surface receptors (CD64) are currently widely utilized as biomarkers for monitoring sepsis [14]. However, these markers lack specificity for sepsis and may also be detected in non-infectious processes or conditions unrelated to sepsis [1]. Chen et al. recently discovered that TN, a plasma and tissue protein found in individuals with sepsis or septic shock, is directly linked to the prevalence of sepsis [11]. Further research has revealed that a pathogenic epitope P5-5 within TN, which contains the peptide sequence NDALYEYLRQ, was critical for TN/HMGB1 complex interaction that led to endocytosis of macrophages and reduction in plasma TN levels [11]. In our previous research, we developed a monoclonal antibody against the P5-5 pathogenic epitope using cell fusion. This antibody can accurately diagnose the presence of sepsis and quantitatively monitor its progression in clinical blood samples [14]. Based on this specific mAb, an ic-ELISA was established as a novel method for sepsis detection. Moreover, the anti-P5-5 mAb was also shown to effectively inhibit sepsis progress in mice by recovering macrophage number and increasing the survival of septic mice.

The formation of an antigen–antibody complex is highly sensitive to even minor alterations in the chemical groups or structures on the antigen surface, which can significantly affect the binding affinity of the antibody. This phenomenon raises a critical scientific question in the P5-5 interaction with mAb: Which specific amino acid within the P5-5 epitope is directly recognized and bound by the therapeutic antibody? To address this question, this study employed alanine scanning mutagenesis, systematically mutating each amino acid of the P5-5 epitope to alanine in sequence. ELISA experiments were subsequently conducted to determine which amino acid mutation abrogated antibody binding, thereby identifying the key amino acids essential for antibody recognition. The results demonstrated that Leu4 and Leu8 are the most critical residues for antibody binding; the loss of either residue completely abolished antibody recognition of the epitope. Additionally, mutations in Asp2, Tyr5, Glu6, and Tyr7 partially reduced antibody binding affinity, indicating their involvement in modulating the interaction but not as crucial as Leu4 and Leu8. Interestingly, antigen epitope prediction algorithms identified a minimum antigenic determinant of the LYEYL (Leu-Tyr-Glu-Tyr-Leu) motif, corroborated by molecular docking studies, which highlighted Leu4, Tyr7, and Leu8 as the key residues involved in binding capacity. These predictions align closely with the experimental mutation data, validating the accuracy and consistency of the computational models. Furthermore, structural biology analysis provides valuable insights and guidance for understanding antibody–epitope interactions. More importantly, in this study, the ten important residues of the P5-5 epitope were verified by point mutation and reduced to seven amino acids (2–8 motif, DALYEYL), which greatly facilitated future disease treatment and vaccine development.

The P5-5 epitope represents the most critical pathogenic determinant within the TN protein. Our prior research demonstrated that monoclonal antibodies targeting this epitope exhibit therapeutic potential in sepsis-induced murine models. Therefore, elucidating the key amino acid residues involved in the P5-5 epitope is of considerable importance for the design and production of next-generation recombinant antibodies.

The affinity constant of an antibody is a fundamental parameter used to characterize the interaction between an antigen and an antibody. In this study, the Beatty-improved non-competitive ELISA method was utilized to evaluate both antibody purity and the affinity constant, which is expressed by the following formula: K_aff_ = (n − 1)/{2(n[Abt] − [Ab])} [32,33]. This assay has been widely adopted and is especially suitable for determining the affinity constant between antibodies and immobilized antigens. The measurable range of the antibody affinity constant (K_aff_) using this method generally spans from 10^6^ to 10^10^ L/mol, which is consistent with the commonly reported affinity range for most monoclonal antibodies (10^7^ to 10^9^ L/mol). In conclusion, this assay method is simple, practical, and capable of producing accurate and reliable results [15,16,22,23,32,33,40,41]. Furthermore, it is not limited by the molecular weight of the antigen and exhibits broad applicability.

Recombinant antibodies are generated through the expression of specific genes, resulting in high batch-to-batch consistency and overcoming the limitations associated with traditional hybridoma-based antibody production. Unlike conventional methods, recombinant antibodies are not constrained by low repeatability or genetic drift, which ensures their stable binding to pathogenic targets and lays the foundation for highly specific and sensitive detection in disease diagnosis and monitoring. In addition, instead of high manufacturing costs, prolonged production cycles, and limited flexibility for genetic modification as were associated with traditional hybridoma antibody production, the production of recombinant gene-engineered antibodies only requires the preservation of antibody-encoding genes obtained via phage display technology, without the risk of cell line contamination or mutation-induced degradation. Collectively, this approach ensures high-throughput and consistent manufacture of reliable therapeutic bio-agents for the patients who are in urgent need.

## 5. Conclusions

In this study, the two most critical amino acid residues of the P5-5 pathogenic epitope were identified through alanine scanning for antibody binding. To our best knowledge, this is the first time to report two key residues (4L and 8L) in the P5-5 pathogenic epitope of interaction between therapeutic antibody and antigen. The results demonstrated that minor structural variations in antigens can significantly impact antigen–antibody binding, a process that is vital for both disease research and vaccine development.

Concurrently, this study also prepared a genetically engineered single-chain variable fragment against P5-5, which establishes the basis for future diagnosis and treatment. The sensitivity of our scFv-based ELISA against TN-P5-5 is 62.86 ng/mL. Furthermore, the experiment takes only two hours because the P5-5 antigen has already been precoated and blocked in the ELISA plate. Moreover, our existing ic-ELISA methods may be used to analyze P5-5 epitope samples in PBS, with a target detection range of 78.125 ng/mL to 1.25 μg/mL. The recombinant fusion scFv might be necessary for specific applications. For instance, intricate fusion proteins like antibody–enzyme hybrids or bispecific antibodies necessitated a recombinant approach, as they could not be synthesized with precise stoichiometry through traditional chemical modification methods. Therefore, our work has broad implications for future studies.

## Data Availability

The data that support the findings of this study are available from the corresponding author upon reasonable request.

## Supplementary Materials

The following supporting information can be downloaded at: https://www.mdpi.com/article/10.3390/biom15081100/s1. Figure S1: Purification of anti-TN-P5-5 mAb; Figure S2: Molecular characterization of scFv; Figure S3: Bioinformatics analysis of mAb 12F1 variable regions; Figure S4: The sequencing results alignment of the expression vector pMAL-c2x-linker-12F1-scFv; Figure S5: Affinity determination of 12F1 scFv; Figure S6: 12F1 scFv inhibitory standard curve for TN-P5-5 pathogenic epitope.

## References

[1] Yang X., Pu X., Xu Y., Zhao J., Fang X., Cui J., Deng G., Liu Y., Zhu L., Shao M. (2025). A novel prognosis evaluation indicator of patients with sepsis created by integrating six microfluidic-based neutrophil chemotactic migration parameters. Talanta.

[2] Singer M., Deutschman C.S., Seymour C.W., Shankar-Hari M., Annane D., Bauer M., Bellomo R., Bernard G.R., Chiche J.D., Coopersmith C.M. (2016). The Third International Consensus Definitions for Sepsis and Septic Shock (Sepsis-3). JAMA.

[3] Cui Z., Wang L., Li H., Feng M. (2023). Study on immune status alterations in patients with sepsis. Int. Immunopharmacol..

[4] Delano M.J., Ward P.A. (2016). Sepsis-induced immune dysfunction: Can immune therapies reduce mortality?. J. Clin. Investig..

[5] Zhu C.L., Wang Y., Liu Q., Li H.R., Yu C.M., Li P., Deng X.M., Wang J.F. (2022). Dysregulation of neutrophil death in sepsis. Front. Immunol..

[6] Tseng Y.T., Yu Y.H., Yeh Y.Y., Mai P.C., Huang T.T., Huang C.J., Chau L.K., Chen Y.L. (2025). Femtomolar-level detection of procalcitonin using a split aptamer-based fiber optic nanogold-linked sorbent assay for diagnosis of sepsis. Talanta.

[7] Chun K., Syndergaard C., Damas C., Trubey R., Mukindaraj A., Qian S., Jin X., Breslow S., Niemz A. (2015). Sepsis Pathogen Identification. J. Lab. Autom..

[8] Reddy B., Hassan U., Seymour C., Angus D.C., Isbell T.S., White K., Weir W., Yeh L., Vincent A., Bashir R. (2018). Point-of-care sensors for the management of sepsis. Nat. Biomed. Eng..

[9] Bradley Z., Bhalla N. (2023). Point-of-care diagnostics for sepsis using clinical biomarkers and microfluidic technology. Biosens. Bioelectron..

[10] Qiu X., Lei Y.P., Zhou R.X. (2023). SIRS, SOFA, qSOFA, and NEWS in the diagnosis of sepsis and prediction of adverse outcomes: A systematic review and meta-analysis. Expert Rev. Anti Infect. Ther..

[11] Chen W., Qiang X., Wang Y., Zhu S., Li J., Babaev A., Yang H., Gong J., Becker L., Wang P. (2020). Identification of tetranectin-targeting monoclonal antibodies to treat potentially lethal sepsis. Sci. Transl. Med..

[12] Li J., Bao G., Wang H. (2020). Time to Develop Therapeutic Antibodies Against Harmless Proteins Colluding with Sepsis Mediators?. Immunotargets Ther..

[13] Paterson C.W., Ford M.L., Coopersmith C.M. (2020). Breaking the bond between tetranectin and HMGB1 in sepsis. Sci. Transl. Med..

[14] Wang J., Liu M., Cai Z., Zahid R., Zhang W., Ma D., Li D., Liang Y., Zha L., Zhou Y. (2024). Pathogenic epitope-specific monoclonal antibody-based immunoassay for accurate diagnosis and monitoring of tetranectin in sepsis. Int. Immunopharmacol..

[15] Tang H., Liu H., Gao Y., Chen R., Dong M., Ling S., Wang R., Wang S. (2022). Detection of alphaB-Conotoxin VxXXIVA (alphaB-CTX) by ic-ELISA Based on an Epitope-Specific Monoclonal Antibody. Toxins.

[16] Tang H., Wang Q., Yang M., Jia R., Yuan J., Wang R. (2025). Development of sensitive immunoassay for identification and detection of mu-KIIIA-CTX: Insights into antibody discovery, molecular recognition, and immunoassay. Int. J. Biol. Macromol..

[17] Wang R., Gu X., Zhuang Z., Zhong Y., Yang H., Wang S. (2016). Screening and Molecular Evolution of a Single Chain Variable Fragment Antibody (scFv) against Citreoviridin Toxin. J. Agric. Food Chem..

[18] Wang R., Fang S., Wu D., Lian J., Fan J., Zhang Y., Wang S., Lin W. (2012). Screening for a single-chain variable-fragment antibody that can effectively neutralize the cytotoxicity of the Vibrio parahaemolyticus thermolabile hemolysin. Appl. Environ. Microbiol..

[19] Martviset P., Thanongsaksrikul J., Geadkaew-Krenc A., Chaimon S., Glab-Ampai K., Chaibangyang W., Sornchuer P., Srimanote P., Ruangtong J., Prathaphan P. (2024). Production and immunological characterization of the novel single-chain variable fragment (scFv) antibodies against the epitopes on Opisthorchis viverrini cathepsin F (OvCatF). Acta Trop..

[20] Jalilzadeh-Razin S., Mantegi M., Tohidkia M.R., Pazhang Y., Pourseif M.M., Barar J., Omidi Y. (2019). Phage antibody library screening for the selection of novel high-affinity human single-chain variable fragment against gastrin receptor: An in silico and in vitro study. Daru.

[21] Ren Z., Zhang H., Yang L., Wang Z., Xiong J., Zheng P., Wang J., Jiang H. (2022). Targeted preparation and recognition mechanism of broad-spectrum antibody specific to Aconitum alkaloids based on molecular modeling and its application in immunoassay. Anal. Chim. Acta.

[22] Tang H., Liu H., Chen R., Gao Y., Dong M., Ling S., Wang R., Wang S. (2022). Development of Immunochromatographic Strip for Detection of alphaB-VxXXIVA-Conotoxin Based on 5E4 Monoclonal Antibody. Toxins.

[23] Wang R., Wang J., Liu H., Gao Y., Zhao Q., Ling S., Wang S. (2021). Sensitive immunoassays based on specific monoclonal IgG for determination of bovine lactoferrin in cow milk samples. Food Chem..

[24] Chang W., Fang J., Han S., Sun H., Zhai T., Wang L., Qi X., Xue Q., Wang J. (2025). Screening and identification of LSDV-specific monoclonal antibodies to establish a double-antibody sandwich ELISA for distinguishing LSDV from SPPV and GTPV. Int. J. Biol. Macromol..

[25] Huang J., Lin A., Gu Y., Pan X., Ma X., Qing Y., Li J. (2025). Fluorescence-activated cell sorting-based efficient screening of monensin monoclonal antibodies and applications in lateral flow immunoassay. Talanta.

[26] Liu Y., Jin Z., Sun D., Xu B., Lan T., Zhao Q., He Y., Li J., Cui Y., Zhang Y. (2025). Preparation of hapten and monoclonal antibody of hesperetin and establishment of enzyme-linked immunosorbent assay. Talanta.

[27] Wang J., Sun C., Hu Z., Wang F., Chang J., Gao M., Ye D., Jia Q., Zou H., Willems L. (2024). Development of a novel monoclonal antibody-based competitive ELISA for antibody detection against bovine leukemia virus. Int. J. Biol. Macromol..

[28] Peng Y., Jin Y., Sun D., Jin Z., Zhao Q., He Y., Jiao B., Cui Y., Zhang Y. (2024). Monoclonal antibody-based icELISA for sensitive monitoring fenpyroximate residue by hydrolysis conversion. Talanta.

[29] Wang R., Zhong Y., Wang J., Yang H., Yuan J., Wang S. (2019). Development of an ic-ELISA and immunochromatographic strip based on IgG antibody for detection of omega-conotoxin MVIIA. J. Hazard. Mater..

[30] Yang H., Zhong Y., Wang J., Zhang Q., Li X., Ling S., Wang S., Wang R. (2018). Screening of a ScFv Antibody with High Affinity for Application in Human IFN-gamma Immunoassay. Front. Microbiol..

[31] Chen Y., Feng C., Huang C., Shi Y., Omar S.M., Zhang B., Cai G., Liu P., Guo X., Gao X. (2024). Preparation of polyclonal antibodies to chicken P62 protein and its application in nephropathogenic infectious bronchitis virus-infected chickens. Int. J. Biol. Macromol..

[32] Beatty J.D., Beatty B.G., Vlahos W.G. (1987). Measurement of monoclonal antibody affinity by non-competitive enzyme immunoassay. J. Immunol. Methods.

[33] Wang R., Zeng L., Yang H., Zhong Y., Wang J., Ling S., Saeed A.F., Yuan J., Wang S. (2017). Detection of okadaic acid (OA) using ELISA and colloidal gold immunoassay based on monoclonal antibody. J. Hazard. Mater..

[34] Yang M., Jia R., Liu Y., Tang H., Wu H., Yuan J., Wang R. (2025). High-sensitivity and rapid immunoassays for furosine detection based on monoclonal antibody 1C3. Int. J. Biol. Macromol..

[35] Xu J., Sun J., Lu X., Wang Y., Zhang Y., Sun X. (2023). A highly sensitive fluorescence immunochromatography strip for thiacloprid in fruits and vegetables using recombinant antibodies. Talanta.

[36] Wang F., Liu M., Ma D., Cai Z., Liu L., Wang J., Zhang W., Zhao L., Zhai C., Xu Y. (2023). Dendritic cell-expressed IDO alleviates atherosclerosis by expanding CD4^+^CD25^+^Foxp3^+^Tregs through IDO-Kyn-AHR axis. Int. Immunopharmacol..

[37] Wang F., Liu L., Wang J., Liu M., Zhang W., Zhao L., Zhai C., Xu Y. (2023). Gain-of-function of IDO in DCs inhibits T cell immunity by metabolically regulating surface molecules and cytokines. Exp. Ther. Med..

[38] Dondelinger M., Filee P., Sauvage E., Quinting B., Muyldermans S., Galleni M., Vandevenne M.S. (2018). Understanding the Significance and Implications of Antibody Numbering and Antigen-Binding Surface/Residue Definition. Front. Immunol..

[39] Liang Y., Wang Y., Wang F., Li J., Wang C., Dong J., Ueda H., Xiao Z., Shen Y., Xu Z. (2021). An enhanced open sandwich immunoassay by molecular evolution for noncompetitive detection of Alternaria mycotoxin tenuazonic acid. Food Chem..

[40] Cai P., Wang R., Ling S., Wang S. (2021). A high sensitive platinum-modified colloidal gold immunoassay for tenuazonic acid detection based on monoclonal IgG. Food Chem..

[41] Wang R., Huang A., Liu L., Xiang S., Li X., Ling S., Wang L., Lu T., Wang S. (2014). Construction of a single chain variable fragment antibody (scFv) against tetrodotoxin (TTX) and its interaction with TTX. Toxicon.

