# Supplementary material for: Identification of Two Critical Contact Residues in a Pathogenic Epitope from Tetranectin for Monoclonal Antibody Binding and Preparation of Single-Chain Variable Fragments
Source: Biomolecules. 2025 Jul 30;15(8):1100. doi: 10.3390/biom15081100 (PMC12383646; doi:10.3390/biom15081100)
Supplement: Supplementary file 1 [file biomolecules-15-01100-s001.zip › biomolecules-3695968-supplementary.pdf]

Identification of Two Critical Contact Residues in a Pathogenic Epitope from Tetranectin for Monoclonal Antibody Binding and Preparation of Single Chain Variable Fragment

Figure Legends for supplementary figures:

Figure S1. Purification of anti-TN-P5-5 mAb.

Figure S2. Molecular characterization of scFv.

Figure S3. Bioinformatics analysis of mAb 12F1 variable regions.

Figure S4. The sequencing results alignment of the expression vector pMAL-c2x-linker-12F1-scFv.

Figure S5. Affinity determination of 12F1 scFv.

Figure S6. 12F1 scFv inhibitory standard curve for TN-P5-5 pathogenic epitope.

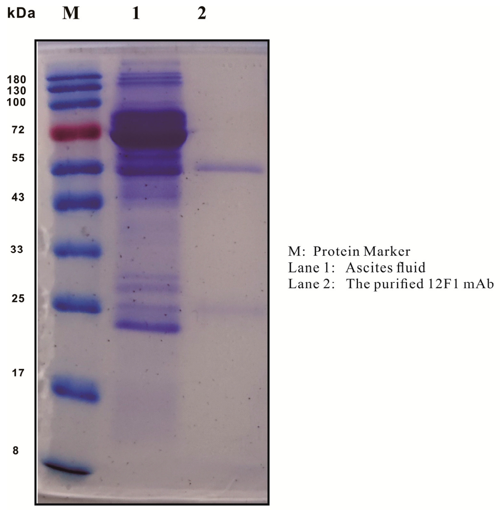

Figure S1. Purification of anti-TN-P5-5 mAb. SDS-PAGE analysis of the purified mAb. Lane M: protein marker, Lane 1: the total protein of induced mice ascites, Lane 2: the purified anti-TN-P5-5 mAb through protein G affinity chromatography.

GAC GTG AAG CTC GTG GAG TCT GGG GGA GGC TTA GTG AAG CTT GGA GGG

D V K L V E S G G G L V K L G G

VH-CDR1

TCC CTG AAA CTC TCC TGT GCA GCC TCT GGA TTC ACT TTC AGT AGC TAT CAC

S L K L S C A A S G F T F S S Y H

ATG TCT TGG GTT CGC CAG ACT CCA GAG AAG AGG CTG GAG TTG GTC GCA

M S W V R Q T P E K R L E L V A

VH-CDR2

GCC ATT AAT AGT AAT GGT GGT AGA ACC TAC CAT TCA GAC ACT GTG AAG GGC

A I N S N G G R T Y H S D T V K G

CGA TTC ACC ATC TCC AGA GAC AAT GCC AAG AAC ACC CTG TAC CTG CAA ATG

R F T I S R D N A K N T L Y L Q M

VH-CDR3

AGC AGT CTG AAG TCT GAG GAC ACA GCC TTG TAT TAC TGT ATA AGA CAC CCC

S S L K S E D T A L Y Y C I R H P

CCT GAT GGG GCC TGG TTT GCT TAC TGG GGC CAA GGG ACT CTG GTC ACT

P D G A W F A Y W G Q G T L V T

Linker

GTC TCT GCA GGT GGA GGC GGT TCA GGC GGA GGT GGC TCT GGC GGT GGA

V S A G G G G S G G G G S G G G

GGA TCG GAT ATC CAG ATG ACA CAG ACT ACA TCC TCC CTG TCT GCC TCT CTG

G S D I Q M T Q T T S S L S A S L

VL-CDR1

GGA GAC AGA GTT ACC ATC AGT TGC AGG GCA AGT CAG GAC ATT AGT AAT TAT

G D R V T I S C R A S Q D I S N Y

TTA AAC TGG TAT CAG CAG AAA CCA GAT GGA ACT GTT AAA CTC CTG ATC TAC

L N W Y Q Q K P D G T V K L L I Y

VL-CDR2

TAC ACA TCA AGA TTA CAC TCA GGA GTC CCA TCA AGG TTC AGT GGC AGT GGG

Y T S R L H S G V P S R F S G S G

TCT GGA ACA GAT TAT TCT CTC ACC ATT AGC AAC CTG GAA CAA GAA GAT ATT

S G T D Y S L T I S N L E Q E D I

VL-CDR3

GCC ACT TAC TTT TGC CAA CAG GGT AAT ACG CTT CCT CCG ACG TTC GGT GGA

A T Y F C Q Q G N T L P P T F G G

GGC ACC AAG CTG GAA ATC AAA

G T K L E I K

Figure S2. Molecular characterization of scFv. Encoding gene and amino acids of scFv. The CDR regions of VH and VL, linker domains are shown with their names adjacent to the domains.

A

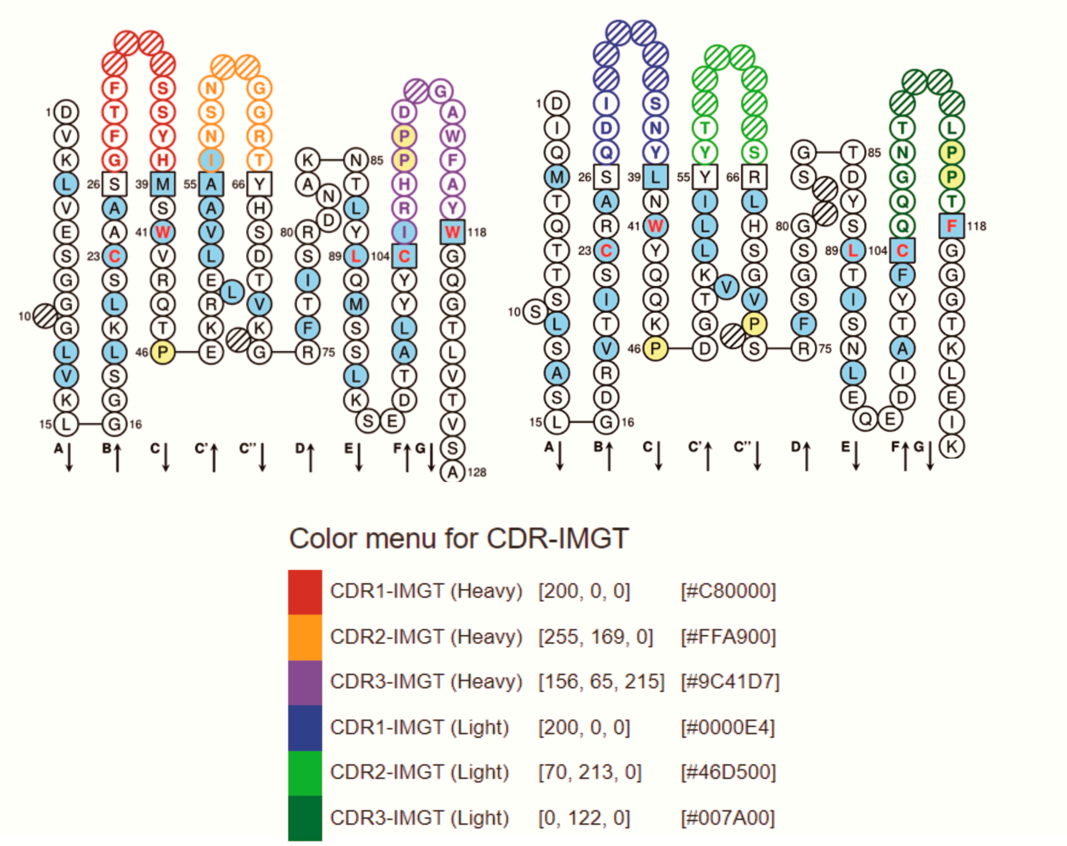

B

**Result summary:** 12F1_VH	Productive IGH rearranged sequence (no stop codon and in-frame junction)		
V-GENE and allele	Musmus IGHV5-6-2*01 F	score = 1404	identity = 98.61% (284/288 nt)
J-GENE and allele	Musmus IGHJ3*01 F	score = 235	identity = 100.00% (47/47 nt)
D-GENE and allele by IMGT/JunctionAnalysis	Musmus IGHD2-3*01 F	D-REGION is in reading frame 3	
FR-IMGT lengths, CDR-IMGT lengths and AA JUNCTION	[25.17.38.11]	[8.8.12]	CIRHPPDGAWFAYW
JUNCTION length (in nt) and decryption	42 nt = (11)0{6}-4(6)-7{0}+2(17)	(3'V)3'{N1}5'(D)3'{N2}5'(5'J)	

**Result summary:** 12F1_VL	Productive IGK rearranged sequence (no stop codon and in-frame junction)		
V-GENE and allele	Musmus IGKV10-96*07 F, or Musspr IGKV10-96*01 F	score = 1368	identity = 98.92% (276/279 nt)
J-GENE and allele	Musmus IGKJ1*01 F	score = 170	identity = 100.00% (34/34 nt)
FR-IMGT lengths, CDR-IMGT lengths and AA JUNCTION	[26.17.36.10]	[6.3.9]	CQQGNTLPPTF
JUNCTION length (in nt) and decryption	33 nt = (26)0{0}-3(7)	(3'V)3'{N}5'(5'J)	

Figure S3. Bioinformatics analysis of mAb 12F1 variable regions. (A) IMGT collier de perle graphical 2-dimensional representations of the 12F1 mAb VH and VL regions. The heavy (VH) and light (VL) chain CDRs are defined by canonical anchor residues (boxed) within adjacent framework regions (FR-IMGT). Sequence analysis was performed through IMGT/V-QUEST, VH CDR1 (red), CDR2 (orange), CDR3 (purple); VL CDR1 (blue), CDR2 (light green), CDR3 (dark green). (B) The VH and VL region homology analysis of 12F1 mAb. The gene combination pattern of VH region of the antibody is V-D-J, while that of the VL region is V-J.

The sequencing results alignment of the expression vector pMAL-c2x-linker-12F1-scFv in TB1:

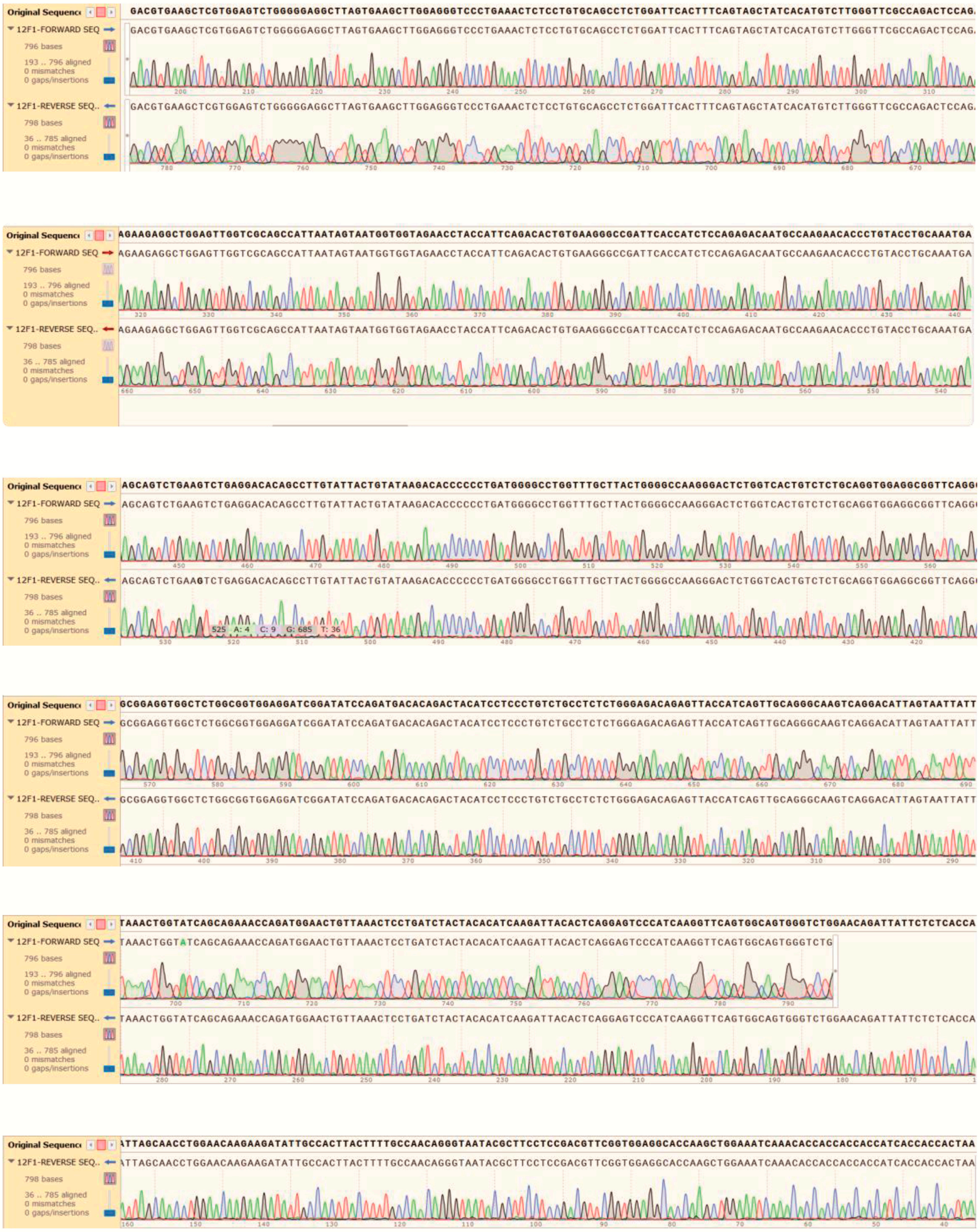

Figure S4. The sequencing results alignment of the expression vector pMAL-c2x-linker-12F1-scFv.

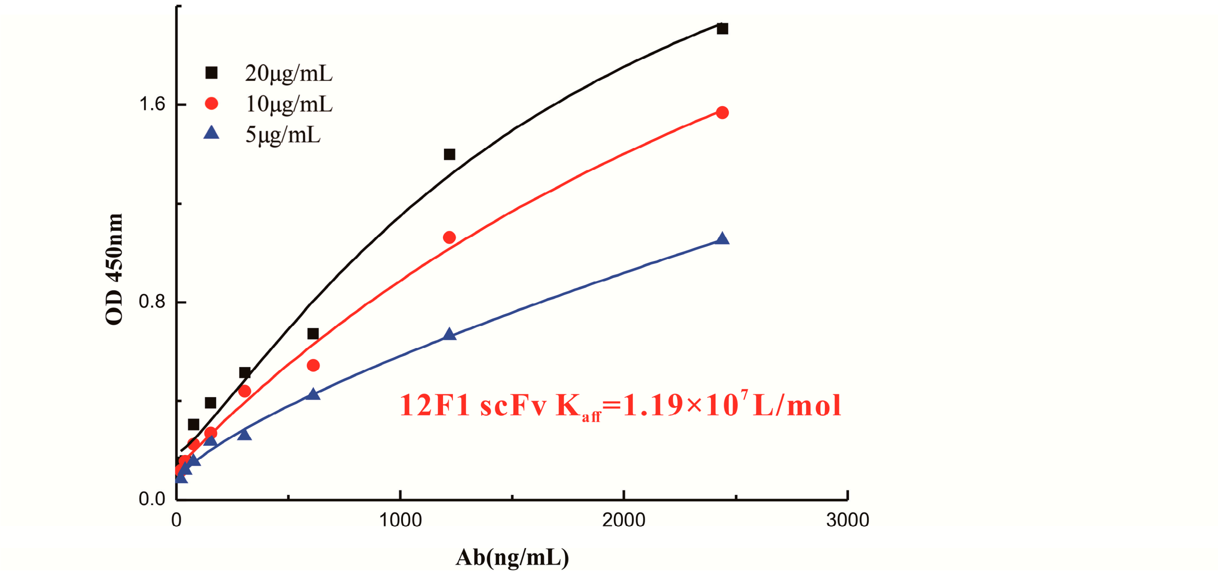

Figure S5. Affinity determination of 12F1 scFv. The affinity constant of the scFv was quantitatively determined through indirect enzyme-linked immunosorbent assay (iELISA) employing a standardized formula-based calculation. Serial concentrations of the coated antigen (20, 10, and 5 μg/mL) were utilized to assess the scFv binding affinity across a range of dilutions (1:50, 1:100, 1:200). Kaff=1.19×10^7 L/mol, belonging to high affinity antibody.

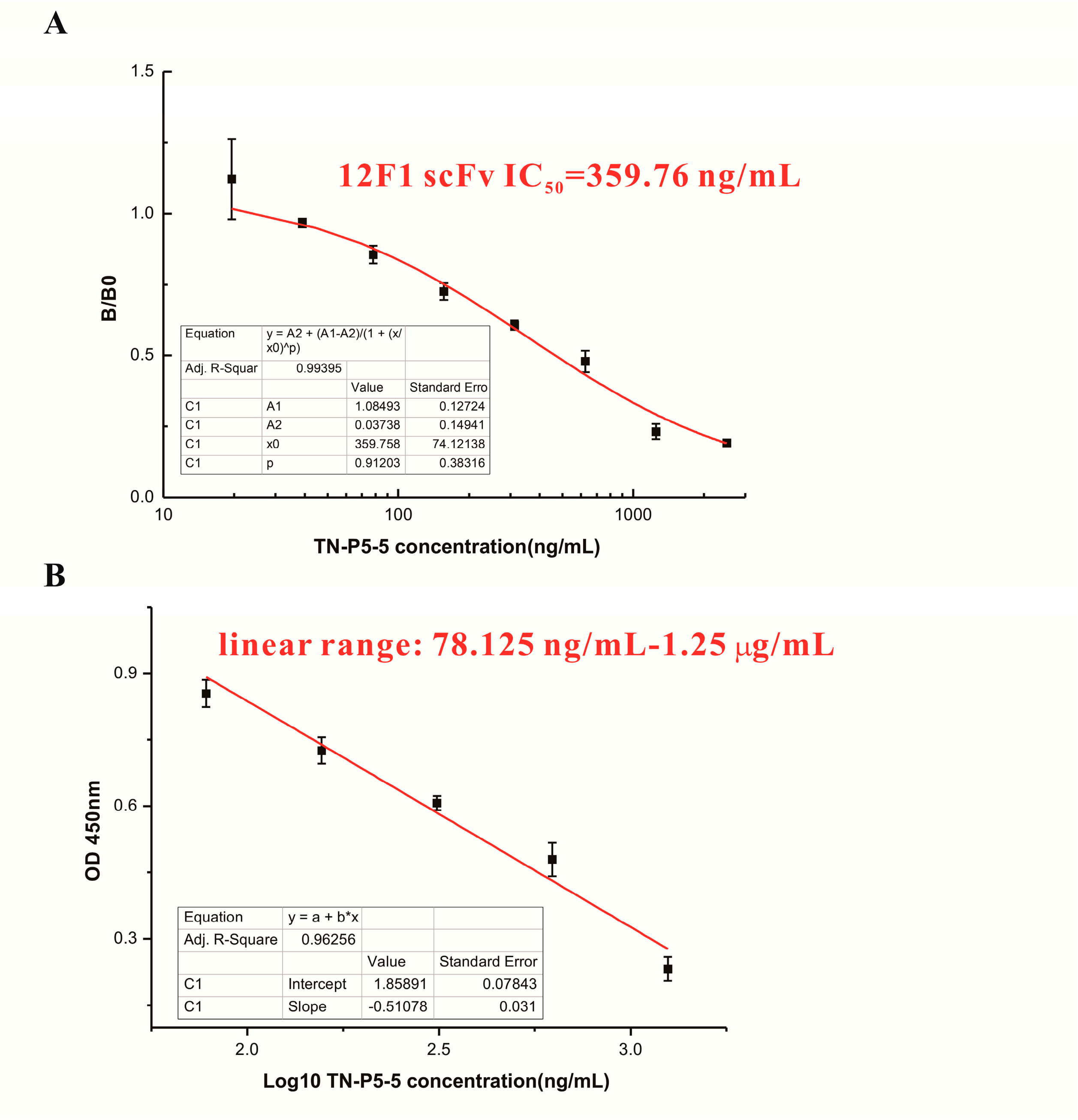

Figure S6. 12F1 scFv inhibitory standard curve for TN-P5-5 pathogenic epitope. (A) A typical calibration curve illustrated by plotting (B/B0) against P5-5. The IC_{50} of TN-P5-5 was 359.76 ng/mL and the limit of detection was 62.86 ng/mL（IC_{90}）. (B) The linear detection range existed from the S-shaped curve, with the equation being y=1.85891-0.51078x, R2=0.96256, with a target detection range of 78.125 ng/mL to 1250 ng/mL.
